# The Faculty of Language Integrates the Two Core Systems of Number

**DOI:** 10.3389/fpsyg.2017.00351

**Published:** 2017-03-16

**Authors:** Ken Hiraiwa

**Affiliations:** ^1^Department of English, Meiji Gakuin UniversityTokyo, Japan; ^2^Department of Linguistics and Philosophy, Massachusetts Institute of TechnologyCambridge, MA, USA

**Keywords:** natural language, number, natural numbers, numerals, core systems of number, grammatical number, syntax, linguistics

## Abstract

Only humans possess the faculty of language that allows an infinite array of hierarchically structured expressions (Hauser et al., 2002; Berwick and Chomsky, 2015). Similarly, humans have a capacity for infinite natural numbers, while all other species seem to lack such a capacity (Gelman and Gallistel, 1978; Dehaene, 1997). Thus, the origin of this numerical capacity and its relation to language have been of much interdisciplinary interest in developmental and behavioral psychology, cognitive neuroscience, and linguistics (Dehaene, 1997; Hauser et al., 2002; Pica et al., 2004). Hauser et al. (2002) and Chomsky (2008) hypothesize that a recursive generative operation that is central to the computational system of language (called *Merge*) can give rise to the successor function in a set-theoretic fashion, from which capacities for discretely infinite natural numbers may be derived. However, a careful look at two domains in language, grammatical number and numerals, reveals no trace of the successor function. Following behavioral and neuropsychological evidence that there are two core systems of number cognition innately available, a core system of representation of large, approximate numerical magnitudes and a core system of precise representation of distinct small numbers (Feigenson et al., 2004), I argue that grammatical number reflects the core system of precise representation of distinct small numbers alone. In contrast, numeral systems arise from integrating the pre-existing two core systems of number and the human language faculty. To the extent that my arguments are correct, linguistic representations of number, grammatical number, and numerals do not incorporate anything like the successor function.

## Introduction

Only humans possess the faculty of language that allows an infinite array of hierarchically structured expressions (Chomsky, 1995; Miyagawa et al., 2013, 2014; Berwick and Chomsky, 2015). Similarly, humans have a capacity for infinite natural numbers, while all other species seem to lack such a capacity (Hauser et al., 2002; Chomsky, 1982, 1986; for studies on the capacity for number, see Gelman and Gallistel, 1978; Wynn, 1992a,b; Dehaene, 1993, 1997; Butterworth, 1999; Pica et al., 2004). This unique capacity is obviously what has made the development of sophisticated mathematics possible (Hauser and Watumull, in press). Common to both faculties is the use of finite means to achieve discrete infinity, that is, an open-ended array of discrete expressions (von Humboldt, 1836; Chomsky, 1965, 2007a,b, 2008, 2010). Thus, the origin of this numerical capacity and its relation to language have been of much interdisciplinary interest in developmental and behavioral psychology, cognitive neuroscience, and linguistics (Dehaene, 1997; Hauser et al., 2002; Pica et al., 2004; Gelman and Butterworth, 2005).

Some developmental psychologists have suggested that the concepts of natural numbers are innate to humans (Gelman and Gallistel, 1978; Wynn, 1992b; Dehaene, 1997). Chomsky (2008, p. 139) hypothesizes that Merge can give rise to the successor function (i.e., every numerosity N has a unique successor, N + 1) in a set-theoretic fashion (1 = one, 2 = {one}, 3 = {one, {one}}, …) and that the capacity for discretely infinite natural numbers may be derived from this. Merge is central to the generative system of language (Chomsky, 2008). It is a set-theoretic recursive combinatorial operation that takes two objects X and Y and forms {X, Y} (e.g., two items *the* and *dog* are combined to form a set {*the, dog*}, which can then be further combined with another item *saw* to form a set {*saw*, {*the, dog*}}). “Operating without bounds, Merge yields a discrete infinity of structured expressions” (Chomsky, 2007a, p. 5). Hauser et al. (2002, p. 15) also suggest that “in parallel with the faculty of language, our capacities for number rely on a recursive computation.”

In natural languages, number most clearly emerges in two domains: grammatical number (Corbett, 2000 and references therein) and numerals (Stampe, 1976; Corbett, 1977; Greenberg, 1978; Comrie, 2005a,b; Kayne, 2010). As I will show, however, representations of number in natural languages do not reveal any straightforward trace of the successor function. This leads us to a central question of this article: how natural numbers are linguistically represented and how such representations are related to other cognitive systems, if any.

There has been much behavioral and neuropsychological evidence that there are two core systems of number cognition that are innately available (Xu and Spelke, 2000; Carey, 2001, 2009; Xu, 2003; Feigenson et al., 2004; McCrink and Wynn, 2004). The first is a system of approximate representation of numerical magnitude, which allows one to compare and discriminate large, approximate numerical magnitudes. The second is a system of precise representation of distinct small numbers: 1, 2, 3, possibly 4. This system allows one to compare and discriminate small numbers of individuals. The analog approximate-magnitude system has a ratio limit of 1:2. Experiments show that 6-month-old infants can discriminate numerosities of 8 and 16 and numerosities of 16 and 32, where the ratio is 1:2, but they fail to discriminate numerosities of 8 and 12 and of 16 and 24, where the ratio is 2:3 (Xu and Spelke, 2000; Barth et al., 2003; Xu, 2003; Feigenson, 2007). In contrast, the small-numbers system has a set size limit of 3 or 4. Experiments show that 10- and 12-month-old infants can identify the larger of 1 and 2 and the larger of 2 and 3, while they fail to discriminate between large numbers (Starkey and Cooper, 1980; Feigenson et al., 2002a,b; Xu, 2003; Carey, 2004; also Barner et al., 2008).

I propose that the grammatical-number system and the numeral system constitute linguistic evidence for these two core systems of number representation. The grammatical-number system reflects the system of precise representation of distinct small numbers alone. In contrast, the numeral system has arisen by integrating the two pre-existing core systems with the recursive combinatorial computation Merge, which is unique to the human language faculty.

(1) a. Grammatical number reflects the core system of precise representation of distinct small numbers alone.
   b. Numeral systems reflect both of the core systems of number and Merge.

Consequently, the concepts of natural numbers and its realization in language are distinct and language interfaces with the two core systems of number.

## Numerical notations: a simple example of the core system of precise representation of small numbers

Around the world, natural numbers have often been represented by numerical-notation systems (Menninger, 1969; Ifrah, 1985, 2000). Dehaene (1997, p. 54) observes that many numerical notation systems denote the first three or four numbers by a specific analog number of identical marks, and the following numbers by essentially arbitrary symbols (e.g., I, II, III, IV, V and 一, 二, 三, 四, 五 in Roman and Chinese/Japanese notations for “1”–“5”). He argues that the limit to “3” or “4” follows from the core system of precise representation of distinct small numbers. It would not be expected if numerical notations reflected the successor function.

## Grammatical number also reflects the core system of precise representation of small numbers

If the two core systems of number are innate to humans, one also expects to find some similar trace of these systems within natural-language syntax.

*Grammatical number* is the grammatical coding of numerical quantity. Some languages overtly mark number on nouns (e.g., *I saw the dog* (singular) vs. *I saw the dog-s* (plural) in English). According to Corbett (2000), grammatical-number systems only come in three varieties. English represents the most common, a singular-plural system that distinguishes “1” and “more than 1.” The second most common system is a singular-dual-plural system, which distinguishes “1,” “2,” and “more than 2” (as in Hopi; see Hale, 1997). Finally, a trial system, although quite rare cross-linguistically, involves a four-way distinction, singular-dual-trial-plural, distinguishing “1,” “2,” “3,” and “more than 3” (as in Larike; see Laidig and Laidig, 1990; Corbett, 2000).

A “quadral” system, in which the precise cardinalities 1 through 4 are all distinguished, is reported but quite controversial: according to Corbett (2000, p. 30) and Dixon (2010), the “quadral” number in such systems should actually be analyzed as paucal (i.e., denoting an approximate, relatively small cardinality), rather than literally quadral. Furthermore, there is no attested case of a “quintal” system or a “discretely infinite” grammatical-number system. Grammatical-number systems never go beyond the limit of 3 (Greenberg, 1978; Hurford, 1987).

Such a limit in natural languages may pose a mystery, given humans' capacity for natural numbers and discrete infinity. What explains this absence? The answer that I am proposing here is that grammatical number is founded on the core system of precise representation of small numbers rather than on the successor function.

(2) Grammatical number reflects the core system of precise representation of small numbers.

With (2), we can understand why grammatical-number systems in natural language fall within the range of 1 (singular) to 3 (trial) and do not go beyond.

## Numeral systems: the two core systems of number + merge

Humans are unique in having evolved to deal with discretely infinite natural numbers, beyond the limits of the two core systems of number (precise representation of small numbers and approximate representation of numerical magnitudes). In contrast with grammatical number, numeral systems in many natural languages do in fact show the distinctive property of discrete infinity.

*Numerals* (or *number words*) are what we often use to count in natural language: they are the linguistic representation of discrete numbers (e.g., *one, two, three*, in English). A numeral usually takes the form of a word or a phrase. It is surprising that all the natural languages, across genetic families and across geographical areas, have come to have numerals: “Every language has a numeral system of finite scope” (Greenberg, 1978, p. 273, Universal #1) (see Nevins et al., 2009 for a rebuttal of the claim in Everett, 2005 that Pirahã lacks numerals). It is even more impressive that the internal composition of numerals (and the abstract arithmetic computations behind this composition) shows distinctive shared structural properties in different languages (Greenberg, 1978; Ionin and Matushansky, 2006; Kayne, 2010; Watanabe, 2010).

I propose that the numeral system emerges when the recursive combinatorial operation Merge integrates the two pre-existing core systems of number. This hypothesis needs to clarify the role played by each of the two core systems and the Merge operation. Let us take them one by one in this section and the next.

### Lower numerals as a reflex of the core system of precise representation of small numbers

While numeral systems in English and Japanese show discrete infinity, it is known that some languages only have a highly restricted set of numerals. Botocudo, a Macro-Ge language in Brazil, only has one numeral, namely “1” (Greenberg, 1978). Aiom, one of the languages spoken in Papua New Guinea, and Walbiri, an indigenous language spoken in Central Australia, only have two numerals, “1” and “2” (Aufenanger, 1960; Hale, 1975).

Significantly, Greenberg (1978, p. 276, Universal #6) makes the following observation: “The largest value for L with systems with only simple lexical representation is 5 and the smallest is 2,” where L is the next largest natural number after the largest expressible in the system. Thus, in natural languages whose numeral system lacks an additive operation, numerals can only go up to “4” (i.e., the numeral system can only distinguish “1,” “2,” “3,” “4,” and “many”).

This limit naturally follows from the core system of precise representation of small numbers.

(3) Lower numerals reflect the core system of precise representation of small numbers.

### Neither one word nor an infinite number of words

A number of natural languages have developed numeral systems that go well beyond the limits of a few small numerals. English and Japanese, for example, have potentially infinite numerals. But when we say so, it does not mean that these languages have a potentially infinite number of arbitrary lexical items corresponding to each natural number. It would be extremely inefficient to use as many different numeral words as there are natural numbers, in which case counting up to “1,000” would require one thousand different arbitrary numeral words. Such a system would also be impossible for children to acquire.

It is even more important to note, however, that no numeral system in any natural language shows any formal (syntactic or morphological) trace of the successor function, even though the natural numbers are generally defined in terms of the successor function (Hauser et al., 2002; Chomsky, 2008; Izard et al., 2008, and references therein). For example, there is no language that has a numeral “15” that is composed by repeating a single numeral “1” 15 times.

(4) A hypothetical numeral “15”
     {1, {1, {1, {1, {1, {1, {1, {1, {1, {1, {1, {1, {1, {1, {1}}}}}}}}}}}}}}}
     (pronounced *one-one-one-one-one-one-one-one-one-one-one-one-one-one-one*)

Such a numeral system would not be usable for natural language.

### Numerical bases as a reflex of the core system of approximate representation of numerical magnitudes

Natural language finds an ingenious solution. Let us consider, for example, natural languages that have a decimal numeral system (e.g., English and Japanese). In such languages, the numerals “1” through “10” are simplex numeral words. But they never continue with a new numeral word for each number beyond 10: instead, they combine (multiples of) numerical bases (e.g., digit numbers “10,” “100,” “1,000,” and so on) and small numerals (from “1” up to the smallest numerical base) (Hurford, 1975; Ionin and Matushansky, 2006; Kayne, 2010).

The invention of numerical bases plays a crucial role in going beyond small numbers. But how did numerical bases come to be a part of natural languages? They are known to show syntactic properties distinct from simplex numerals (e.g., *hundreds vs*. ^*^sevens, two hundred vs. ^*^two seven; Corbett, 1977; Kayne, 2010). A crucial fact is that numerical bases and multiples/powers of numerical bases (so-called round numbers), in contrast with simplex numerals, can refer not only to exact numbers like “10,” “100,” and “1,000,” but also to vague/approximate magnitudes (e.g., *a hundred/thousand/million thanks, a hundred/ thousand/million things to do*; similarly, in Japanese, numerical bases, such as *hyaku* “100,” *sen* “1,000,” *man* “10,000,” can mean “numerous/many”) (see also Dehaene, 1997; Krifka, 2002; but see also Musolino, 2004 and references therein for discussions on a different kind of “non-exact” interpretation of numerals “at least *n*” and “at most *n*”). Thus, a natural candidate for the origin of such “analog” numerical bases in natural language is the core system of representation of approximate magnitudes.

(5) Numerical bases in numeral systems reflect the core system of approximate representation of numerical magnitude.

Thus, the core system of precise representation of small numbers is the basis for the simplex numerals in natural language, while the core system of approximate representation of numerical magnitudes is the basis for numerical bases.

There are two remaining questions here. First, a set of small simplex numerals and a set of numerical bases are not sufficient to give rise to discrete infinity (Feigenson et al., 2004; Izard et al., 2008). Second, we have to answer why other species apparently cannot go beyond the limits of these two core systems and humans can.

## 1, 2, 3,…Infinity!

Feigenson et al. (2004, p. 313) speculate that having the two core systems of number enabled humans to go beyond the limits of these two systems' representations with each other. However, these two core systems are shared by other, non-human species (Dehaene, 1997; Brannon and Terrace, 1998; Carey et al., 2000; Carey and Hauser, 2003; Hauser et al., 2003; Feigenson et al., 2004). Somehow, quite a few natural languages have come to employ the finite means supplied by these two systems (lower numerals and numerical bases) to obtain higher numerals, virtually with discrete infinity. Then, there must be something uniquely human that integrates these two systems, allowing discretely infinite higher numerals to be generated.

This is where the third factor—the recursive and combinatorial operation Merge—comes into play. In addition to lower numerals and numerical bases, higher numerals in natural language make a crucial use of the additive operation, in the form of conjunction (*and* in English; *ne* in Dagaare, a language spoken in Ghana; and a covert conjunction in Japanese) (see also Hauser, 2009).

(6) four hundred *and* forty-two (people) (4 × 100 & 4 × 10 (&) 2
     (people))                                                                  (English)
     (noba) kɔɔre anaare *ne* lezaε ayi *ne* bayi (100 × 4 & 20 × 2 & 2
     (people))                                                                  (Dagaare)
     yon-hyaku yon-zyuu ni (nin) (4 × 100 (&) 4 × 10 (&) 2
     (people))                                                   (Japanese)

**Figure 1:**
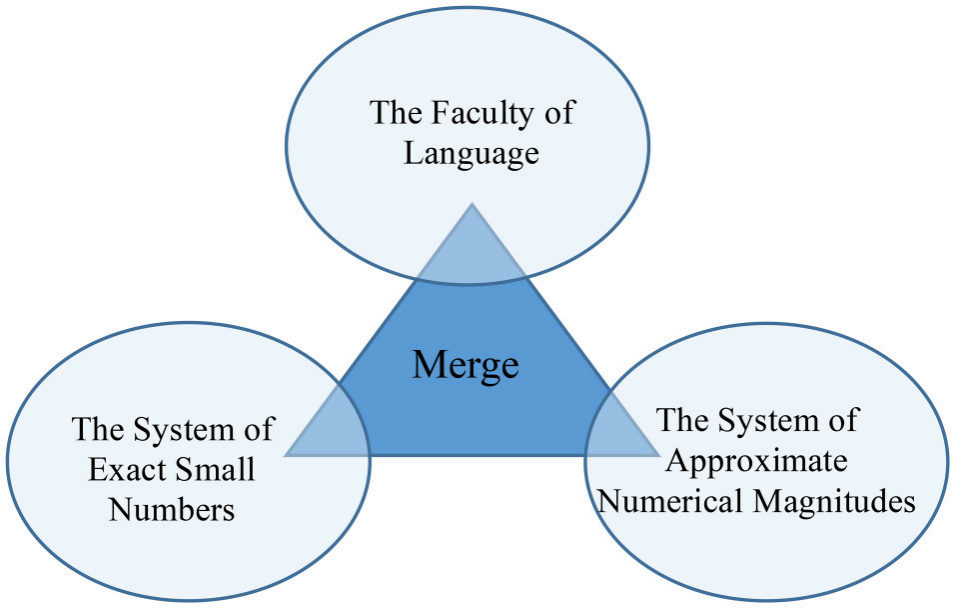
**Integration of the two core systems of number and the faculty of language**.

But numerals are acquired by children long before they learn basic arithmetic, including addition and subtraction. In other words, the additive operation in natural language syntax comes free. But how? I concur with Hauser et al. (2002) that the recursive combinatorial computation Merge plays a crucial role in discrete infinity in natural language (see also Watumull et al., 2014). With Merge available only to humans, the additive operation “A & B” uniquely comes free in the form of conjunction. Thus, it is Merge that integrates the two pre-existing core systems of number (simplex numerals and numerical bases, respectively) and generates an open-ended list of hierarchically structured complex numerals by combining simplex numerals and numerical bases (Figure 1).

(7) The numeral system = the two core systems of number + Merge

This is a feature shared by all natural languages with higher numeral systems. It explains why non-human species can deal with distinct small numbers and approximate numerical magnitudes, but cannot go beyond the limits set by these systems to attain discrete infinity. Humans overcome this problem with the faculty of language, specifically, Merge. By conjoining distinct small numerals with large numerical bases recursively (e.g., *forty thousand, four hundred and forty-two*), natural language can represent a potentially infinite number of numerals. (The same goes for the numerical notations discussed earlier, which use a similar compositional principle.)

From this point of view, we can also understand why there are such languages as Mundurucú and Pirahã that have a very limited number of numerals (Gordon, 2004; Pica et al., 2004). It is likely that such a restricted numeral system resulted from deploying the core system of precise representation of small numbers alone, instead of deploying Merge to integrate both core systems of number. Therefore, it ends up with the first few numerals (1–3 or 1–4) and anything beyond is represented as “many”—much like in grammatical-number systems. Consequently, Mundurucú, and Pirahã lack a principled numeral system (or a counting list) that composes numerals combinatorially, recursively, and systematically, which is crucial for understanding the notion of a successor function (Pica et al., 2004; Izard et al., 2008; Pica and Lecomte, 2008; see also Gelman and Gallistel, 1978; Carey, 2001, 2004; Le Corre and Carey, 2007; Condry and Spelke, 2008).

## Conclusion

The grammatical-number system in natural language deploys the core system of precise representation of distinct small numbers alone. The full numeral system in natural language deploys both of the core systems of number, but it is only made possible by integrating the two core systems of number with the central combinatorial operation Merge of the faculty of language. While the idea that language is closely connected to children's understanding of number words is not new (Carey, 2001, 2004; Condry and Spelke, 2008), the present study explicates what specific function language plays in deriving discretely infinite numerals.

To the extent that my arguments are correct, representations of number systems in natural language do not incorporate anything like the successor function, although abstract computation for the concepts of natural numbers perhaps does (Hale, 1975, p. 296; Hauser et al., 2002; Chomsky, 2008; Izard et al., 2008; Watanabe, in press). This gap between the concepts of natural numbers (in the sense of the successor function) and the linguistic representation of number suggests that they are not in direct correspondence (Gelman and Butterworth, 2005).

My claim that the faculty of language integrates the two core systems of number explains three things. First, numeral systems are not innate, nor do they come free. They are subject to learning (Fuson, 1988; Wynn, 1992b) and allow for variation. Second, no number systems manifest the successor function linguistically. Finally, all natural languages with a full numeral system compose hierarchically structured numerals by conjoining simplex numerals and numerical bases. The picture that emerges from this study is one in which the faculty of language interfaces with the language-external core systems of number.

## Author contributions

This article is single-authored by KH.

### Conflict of interest statement

The author declares that the research was conducted in the absence of any commercial or financial relationships that could be construed as a potential conflict of interest.
